# Platelet Parameters in Hepatic Hydatid Cysts

**DOI:** 10.1155/2013/593273

**Published:** 2013-05-27

**Authors:** Mustafa Sit, Gülali Aktaş, Edip Erdal Yilmaz, İsmail Necati Hakyemez, Aytekin Alçelik, Abdülkadir Küçükbayrak

**Affiliations:** ^1^Abant Izzet Baysal University Hospital, Department of General Surgery, 14280 Bolu, Turkey; ^2^Abant Izzet Baysal University Hospital, Department of Internal Medicine, 14280 Bolu, Turkey; ^3^Abant Izzet Baysal University Hospital, Department of Infectious Diseases, 14280 Bolu, Turkey

## Abstract

*Background*. Hepatic hydatid cyst infection is caused by microorganisms named *Echinococcus* which belong to family Taeniidae. Platelets are considered as a mediator in inflammation and infectious diseases because of the various proinflammatory substances that they contain. *Design and Methods*. Thirty-three patients who were admitted to Doğubayazıt State Hospital's General Surgery Clinic with a diagnosis of hepatic cyst hydatid were enrolled in this retrospective study. Laboratory data of the patients in pre- and postoperative periods were obtained from computerized medical records database of the hospital. *Results*. Preoperative mean platelet volume (MPV) of the patients was significantly increased compared to postoperative MPV values. *Conclusion*. We claim that MPV is a useful follow-up marker after surgery in patients with hydatid cyst.

## 1. Introduction

Hepatic hydatid cyst is caused by microorganisms named *Echinococcus* which belong to family Taeniidae. The infection gives rise to cysts formations in intermediate hosts. These cysts are named as hydatid cysts. Although hydatid cysts may occur in any internal organs, they usually develop in lungs and liver. The disease is one of the most important public health issues in developing and underdeveloped regions of the world. Size, location, and manifestation of the cysts are the basis of the treatment decision. The conventional treatment option is surgery. Surgeons should be very cautious, because rupture of the cysts may occur as a complication in surgery. In selective cases, medical treatment is another treatment option.

Platelets are the smallest cells in blood with a biconvex discoid shape, but they are not typical cells as they lack nucleus. When they are activated, their shape changes and they develop long pseudopods. They are considered as a mediator in inflammation because they contain various proinflammatory substances. It has been shown that platelets have ability to release bactericidal substances and are capable of engaging and encapsulating microorganisms. Furthermore, they are effective defenders in helminthic infections. Joseph et al. [1] reported that platelets can eliminate parasites independent of white blood cells.

Because old platelets shrink, an increase in releasing of young platelets from bone marrow causes an elevation in mean platelet volume (MPV) value as seen in conditions associated with increased platelet production and/or destruction. Expression of CD62P and CD63 and concentration of beta thromboglobulin and platelet factor 4, which reflect platelet activation, have been shown to increase in echinococcosis. Another indicator for platelet activation is MPV. 

We aimed in this study to evaluate pre- and postoperative MPV values of the patients with hepatic hydatidosis.

## 2. Design and Methods

Thirty-three patients who were admitted to Doğubayazıt State Hospital's General Surgery Clinic with a diagnosis of hepatic cyst hydatid were enrolled in this retrospective study. All patients have undergone surgery. Laboratory data of the patients in preoperative period and the postoperative 30th day were obtained from computerized medical records database of the hospital. Hemoglobin level (Hb), hematocrit (Htc), platelet count (PLT), mean platelet volume (MPV), and platelet distribution width (PDW) values were recorded in both pre- and postoperative periods. Data were expressed as mean ± standard deviation.

Blood samples were stored into sterile standard tubes containing constant amount of anticoagulant. The complete blood count analyses were performed by automatic analysers of LH 780 model of Beckman Coulter (Beckman Coulter Inc.; Brea CA, USA). Original kits of the product were used in analyses. 

Data were analyzed using the SPSS for Windows 15.0, Inc., Chicago, IL, USA. Paired samples *t-*test was used for evaluating the difference between pre- and postoperative hemogram parameters. Statistical significance was set for a *P* value of <0.05. The study was approved by the local ethics committee of Abant Izzet Baysal University School of Medicine.

## 3. Results

We enrolled 33 patients who had undergone hydatid cyst surgery in the General Surgery Clinic of Doğubayazıt State Hospital. Twenty-four (%74) patients were female and nine (%26) were male. Age of the patients ranged from 21 to 70 years (mean age: 45 years). Hemoglobin, hematocrit levels, and platelet count were not significantly different in pre- and postoperative periods. In terms of platelet parameters, PDW was not significantly different but preoperative MPV was significantly increased compared to postoperative MPV values (*P* < 0.0001). Table 1 shows preoperative and postoperative laboratory parameters of the patients. 

## 4. Discussion

As they are major actors in hemostasis, platelets are also important mediators in inflammation. Nachman and Weksler [2] reported that platelets were degranulated in bacterial and viral infections. There are also several studies in literature indicating that platelets have antimicrobial effects. Furthermore, Polack et al. [3] reported that platelets have cytotoxic effects against parasites both *in vivo* and *in vitro*. These findings suggest that platelets are involved in infectious diseases. 

Platelet parameters, such as MPV, are associated with parasitic infections. Catal et al. [4] found that MPV is significantly higher in patients with upper urinary tract infection. In Ladhani et al.'s study [5], authors observed platelet parameters in patients with malaria and compared them to subjects without malaria. They found that MPV was significantly increased in patients with malaria than in subjects without malaria. Similarly, Fajardo and Rao [6] detected an increase in thrombocyte size in patients with malaria in their study. Recently, Küçükbayrak et al. [7] observed preoperative and postoperative MPV values of patients with pulmonary hydatid cysts and found that MPV values were significantly increased in preoperative period compared to postoperative period. Our results were in accordance with the literature. We found that MPV of the patients with hepatic hydatid cysts in preoperative period was significantly elevated when compared to MPV values in postoperative period.

In conclusion, we claim that MPV is a useful follow-up marker after surgery in patients with hydatid cyst. Because of the relatively small sample size in the present study, our results should be confirmed in other studies with a larger population. 

## Figures and Tables

**Table 1:** Laboratory parameters of the patients (mean ± standard deviation).

Parameter	Preoperative period	Postoperative period	*P* value
Hb (gr/dL)	12.9 (±1.3)	12.8 (±1.7)	0.383
Htc (%)	39 (±3.7)	38.6 (±4.7)	0.512
Plt (cells/mm^3^)	269000 (±68000)	292000 (±93000)	0.237
MPV (fL)	9.26 (±1.5)	8.43 (±1.1)	<0.0001
PDW	13.7 (±3.1)	13.9 (±2.9)	0.507

## List of Abbrevations

Hb: Hemoglobin
Htc: Hematocrit
PLT: Platelet count
MPV: Mean platelet volume
PDW: Platelet distribution width.
